# Local Multi-Channel RF Surface Coil versus Body RF Coil Transmission for Cardiac Magnetic Resonance at 3 Tesla: Which Configuration Is Winning the Game?

**DOI:** 10.1371/journal.pone.0161863

**Published:** 2016-09-06

**Authors:** Oliver Weinberger, Lukas Winter, Matthias A. Dieringer, Antje Els, Celal Oezerdem, Jan Rieger, Andre Kuehne, Antonino M. Cassara, Harald Pfeiffer, Friedrich Wetterling, Thoralf Niendorf

**Affiliations:** 1 Berlin Ultrahigh Field Facility (B.U.F.F.), Max Delbrueck Center for Molecular Medicine in the Helmholtz Association, Berlin, Germany; 2 Experimental and Clinical Research Center (ECRC), a joint cooperation between the Charité Medical Faculty and the Max Delbrueck Center for Molecular Medicine, Berlin, Germany; 3 Berlin Ultrahigh Field Facility (B.U.F.F.), Max Delbrueck Center for Molecular Medicine in the Helmholtz Association, Berlin, Germany; 4 Berlin Ultrahigh Field Facility (B.U.F.F.), Max Delbrueck Center for Molecular Medicine in the Helmholtz Association, Berlin, Germany; 5 Experimental and Clinical Research Center (ECRC), a joint cooperation between the Charité Medical Faculty and the Max Delbrueck Center for Molecular Medicine, Berlin, Germany; 6 Berlin Ultrahigh Field Facility (B.U.F.F.), Max Delbrueck Center for Molecular Medicine in the Helmholtz Association, Berlin, Germany; 7 German Centre for Cardiovascular Research (DZHK), Berlin, Germany; 8 MRI.TOOLS GmbH, Berlin, Germany; 9 Medical Metrology Department, Physikalisch Technische Bundesanstalt (PTB), Braunschweig and Berlin, Germany; 10 Medical Metrology Department, Physikalisch Technische Bundesanstalt (PTB), Braunschweig and Berlin, Germany; 11 Institute of Neuroscience, Trinity College Dublin, Dublin, Ireland; 12 Berlin Ultrahigh Field Facility (B.U.F.F.), Max Delbrueck Center for Molecular Medicine in the Helmholtz Association, Berlin, Germany; 13 Experimental and Clinical Research Center (ECRC), a joint cooperation between the Charité Medical Faculty and the Max Delbrueck Center for Molecular Medicine, Berlin, Germany; 14 MRI.TOOLS GmbH, Berlin, Germany; Shenzhen institutes of advanced technology, CHINA

## Abstract

**Introduction:**

The purpose of this study was to demonstrate the feasibility and efficiency of cardiac MR at 3 Tesla using local four-channel RF coil transmission and benchmark it against large volume body RF coil excitation.

**Methods:**

Electromagnetic field simulations are conducted to detail RF power deposition, transmission field uniformity and efficiency for local and body RF coil transmission. For both excitation regimes transmission field maps are acquired in a human torso phantom. For each transmission regime flip angle distributions and blood-myocardium contrast are examined in a volunteer study of 12 subjects. The feasibility of the local transceiver RF coil array for cardiac chamber quantification at 3 Tesla is demonstrated.

**Results:**

Our simulations and experiments demonstrate that cardiac MR at 3 Tesla using four-channel surface RF coil transmission is competitive versus current clinical CMR practice of large volume body RF coil transmission. The efficiency advantage of the 4TX/4RX setup facilitates shorter repetition times governed by local SAR limits versus body RF coil transmission at whole-body SAR limit. No statistically significant difference was found for cardiac chamber quantification derived with body RF coil versus four-channel surface RF coil transmission. Our simulation also show that the body RF coil exceeds local SAR limits by a factor of ~2 when driven at maximum applicable input power to reach the whole-body SAR limit.

**Conclusion:**

Pursuing local surface RF coil arrays for transmission in cardiac MR is a conceptually appealing alternative to body RF coil transmission, especially for patients with implants.

## Introduction

In current clinical cardiac MR (CMR), integrated large volume body radiofrequency (RF) coils are commonly used for ^1^H excitation together with close-fitting receive-only (RX) RF surface coil arrays [1]. The large-volume excitation with a body RF coil bodes well for a uniform transmission field (B_1_^+^-field) across the upper torso, which is nearly independent of the RF coil’s loading and the target region under investigation. RF energy provided by the body RF coil is deposited over a large volume though. Therefore RF power is limited by the whole-body and partial-body specific absorption rate (SAR) governed by the International Electrotechnical Commission (IEC) guidelines [2]. Unlike body RF coils, local transmit (TX) RF coils are positioned close to the chest, excite spins in a limited region of the upper torso and deposit the majority of the RF energy there. They have to obey local maximum SAR limits averaged over 10 gram tissue (SAR_10g_) [3].

Progress in ultrahigh field MR (B_0_≥7T) demonstrated that local transceiver (TX/RX) RF coil arrays [4–9] are suitable for RF excitation of deep lying regions such as the heart [10]. The degrees of freedom provided by the independent transmit channels improve excitation fidelity and help to manage B_1_^+^-field inhomogeneities induced by wavelength shortening ($\lambda_{myocardium}^{7T}\approx 13\;cm$) by B_1_^+^-shimming techniques [11] or parallel transmission approaches [12, 13]. While most of the effort on transmit-receive array structures for CMR is currently occurring at 7T, recognition of the benefits of these structures may result in an eventual migration to 3T ($\lambda_{myocardium}^{3T}\approx 30\;cm$), where RF inhomogeneities and SAR limitations, though of less extent, remain significant in clinical CMR [14–16]. This is a powerful motivator to elucidate the performance of local multi-channel surface RF coils versus large volume body RF coil transmission for CMR at 3T, which has not been explored comprehensively yet.

Recognizing this opportunity, this work demonstrates the feasibility of cardiac MR at 3T with a local four-channel TX/RX RF coil array [17–19]. This setup is benchmarked against the clinical standard of a large volume body RF coil for excitation in conjunction with (i) a 32-channel RX-only RF coil array or (ii) a four-channel RX-only RF coil array for reception, both being tailored for CMR. Our assessment includes electromagnetic field (EMF) simulations to detail RF power deposition and to explore B_1_^+^-homogeneity performance and transmit efficiency for the local and body RF coil configuration. For both excitation regimes transmission field maps are acquired in a human torso phantom and in healthy subjects. The feasibility of the local TX/RX RF coil array for high spatial resolution 2D steady-state free precession (SSFP) CINE imaging of the heart at 3T is demonstrated in a volunteer study. For this purpose flip angle distributions and blood-myocardium contrast across the heart are examined for each transmission regime together with left ventricular (LV) chamber quantification. It is shown that image quality and LV function obtained with the local transceiver RF coil array are competitive with those derived from the traditional approach using body RF coil transmission. Practical implications and limitations of multi-channel surface RF coil transmission for cardiac MR are discussed.

## Methods

### RF Hardware

This study was conducted with a wide-bore (70 cm) 3T whole-body MR system (MAGNETOM Verio, Siemens Healthcare, Erlangen, Germany) equipped with a gradient system that offers a maximum slew rate of 200 mT/m/ms and a maximum gradient strength of 45 mT/m. Three RF coil configurations were employed:

#### 4TX/4RX: Four-channel local transceiver surface RF coil array

**Fig 1:**
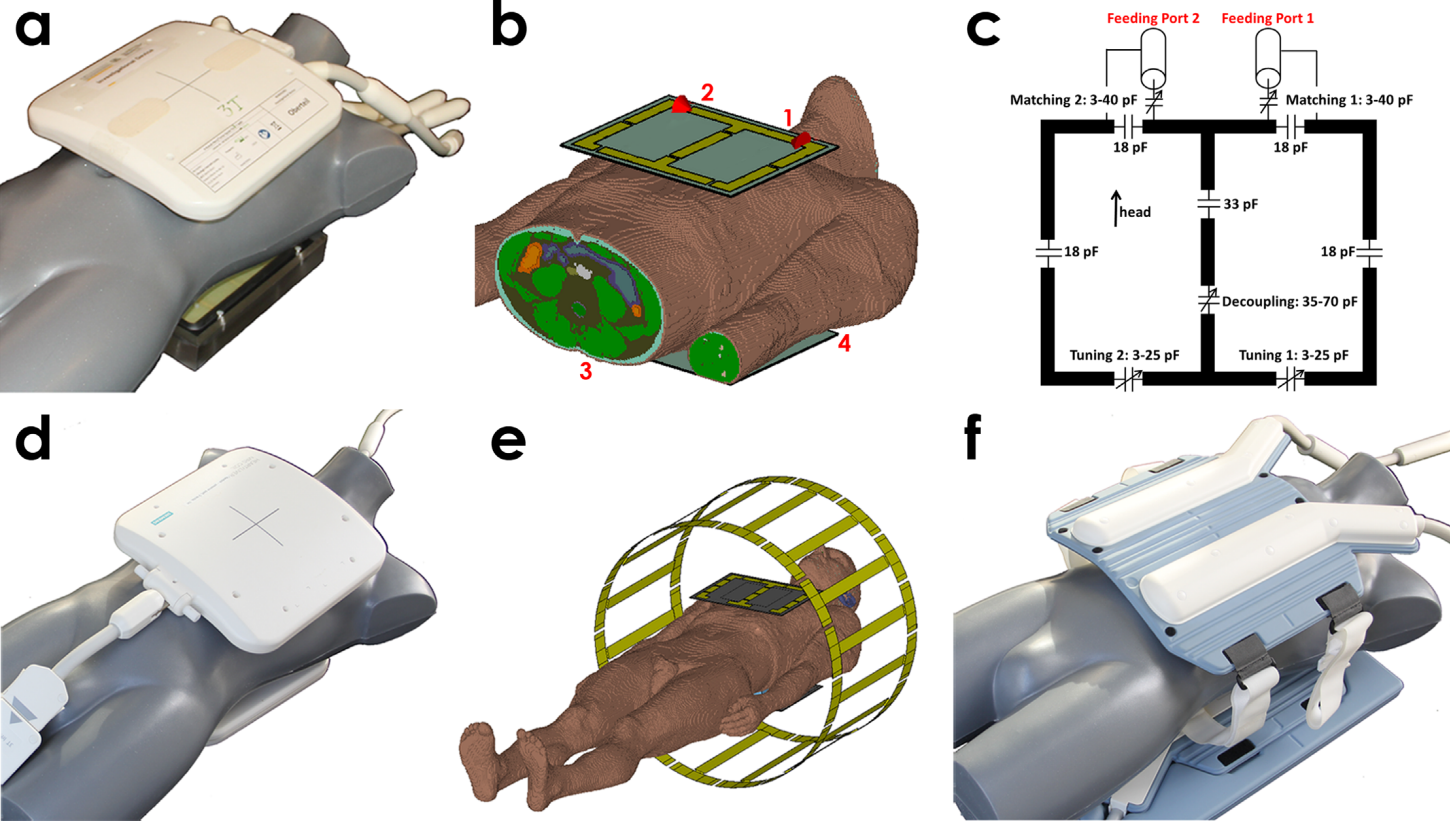
Photographs and simulation setups of the used RF coil configurations. (a) local four-channel TX/RX RF surface coil array (4TX/4RX) and (b) its EMF simulation setup loaded with the truncated human voxel model Duke. The feeding ports of the RF coil are marked in red. (c) basic circuit diagram of the 4TX/4RX RF coil. (d) photograph of the four-channel RX-only RF surface coil (4RX). (e) EMF simulation setup of the body RF coil loaded with the voxel model Duke. The detuned four-channel receive-only surface RF coil (4RX) is included to examine possible field distortions [23]. (f) photograph of the 32-channel RX-only RF surface coil. In configuration (d) and (f) the body RF coil (BC) transmits.

A local transceiver RF coil array (4TX/4RX) comprising four loop elements meeting the needs of patient comfort and ease of clinical use was built (f_0_ = 123 MHz). The four coil loops were constructed on two slightly curved lightweight formers to conform to an average torso. Two pairs of rectangular loops were mounted each on the anterior and the posterior former (Fig 1A–1C). The loop size was set to H-F = 180 mm and L-R = 120 mm to enable whole heart coverage and appropriate depth penetration [20]. Coil decoupling for neighboring loops was achieved by a common conductor with a shared decoupling capacitor (see Fig 1C) [21]. After tuning and matching the largest reflection and coupling parameters of the RF coil, (n = 5 subjects) were S_11_ = -10 dB and S_34_ = -21 dB. The RF transmit power was provided by an RF amplifier (P_max_ = 8 kW, Anaren®, Syracuse New York, USA) and was equally split by hybrid couplers (Stark Contrast, Erlangen, Germany) into four feeding ports highlighted by the red numbers in Fig 1B and 1C. With the four independent feeding ports the transceiver array offers three degrees of freedom for basic B_1_^+^-phase shimming, which was realized by inserting phase shifting cables into the transmit path between the power splitter and the transmit/receive switch (Stark Contrast, Erlangen, Germany).

#### BC/4RX: Large volume TX body RF coil in conjunction with a four-channel RX surface array

For this configuration a large volume body RF coil (BC) was used for transmission (f_0_ = 123 MHz). The body RF coil is a birdcage volume RF coil consisting of 16 equidistant rungs with a total length of 45 cm (Fig 1E). The body RF coil is driven by a 35 kW RF power amplifier at two feeding ports. The RF phase between these ports is fixed resulting in an elliptical polarized excitation without allowing modifications of the B_1_^+^-field distribution. To facilitate high receive sensitivities, signal reception was performed with a home-built four-channel receive-only RF coil array (4RX, f_0_ = 123 MHz). This receive array resembles the geometry of the 4TX/4RX RF coil (Fig 1D). The computer model of the body RF coil together with the 4RX RF coil is shown in Fig 1E.

#### BC/32RX: Large volume TX body RF coil in conjunction with a 32-channel RX surface array

To benchmark the 4TX/RX RF coil against the clinical standard of a state of the art 32-channel receive-only RF coil array (32RX, Invivo, Gainesville, Florida, USA, f_0_ = 123 MHz) was used for reception in conjunction with the body RF coil described above (Fig 1F).

### Electromagnetic Field Simulations

Numerical electromagnetic field (EMF) simulations were performed in CST Studio Suite 2012 (CST AG, Darmstadt, Germany) to estimate B_1_^+^ and SAR distributions of the transmit RF coils. For the body RF coil simulations the entire voxel models Duke (1.74 m, 70 kg, 34 years, BMI: 23.1 kg/m²) and Ella (1.60 m, 58 kg, 26 years, BMI: 22.7 kg/m²) from the Virtual Family [22] were included (Fig 1E). With a total simulation mesh cell number of 34 million for Duke and 30 million for Ella the mesh resolution inside the body models was ~3 mm in the axial plane and ~5 mm along the z-direction. To consider potential interactions a detuned model of the 4RX RF coil was incorporated in the simulations [23].

For EMF simulations of the 4TX/4RX setup the voxel models Duke and Ella were truncated along the head-feet axis (Fig 1B) to save computational time. The total mesh count was 28 and 23 million mesh cells for Duke and Ella, which translates into an isotropic mesh resolution of ~2 mm inside the body models.

For all simulations *k* = max(SAR_10g_)/*P*_in_ was calculated and transferred to the scanner, which uses it to determine the maximum RF input power *P*_*in*_ = *P*_*forward*_−*P*_*reflected*_ for the in vivo measurements according to the IEC guidelines [2].

To avoid dominating local SAR hotspots for the local TX/RX coil, the distance between the RF coil array and the body surface of the human voxel was adapted. For this purpose a distance of 1cm between the body and the RF coil array was realized for the anterior section. For the posterior coil section a distance of 2 cm between the body surface and the RF coil array was accomplished.

### Transmit Phase Settings for the Four-Channel TX/RX RF Coil Array

#### Phase setting Φ_1_

This phase setting was derived from in vivo data obtained for a healthy male subject (54 years, BMI: 24 kg/m²) with a parallel TX system (3T MAGNETOM Verio, Siemens Healthcare, Erlangen, Germany). Relative transmit phase maps [24] of each channel were acquired for a four-chamber view of the heart. The relative transmit phase of each channel was averaged inside a ROI accommodating the heart. The differences of these calculated phase shifts were compensated in order to induce maximal constructive B_1_^+^-interference in the given ROI. Hence **Φ**_1_ maximizes B_1_^+^-efficiency in the heart and does not take B_1_^+^-homogeneity nor local SAR_10g_ considerations into account. **Φ**_1_: *φ*_1_ = 0°, *φ*_2_ = −265°, *φ*_3_ = −92°, *φ*_4_ = −358°

#### Phase setting Φ_2_

Phase setting **Φ**_2_ was derived from EMF simulations using the voxel model Duke and Ella. For this purpose simulated B_1_^+^-fields were extracted for each individual channel for Duke and Ella and interpolated on an equidistant 3 mm grid. A matrix ***A*** was generated containing all *N* voxels of Duke’s and Ella’s heart for all channels resulting in a (*N x 4)* matrix, so that $B_{1}^{+}(\Phi )=A*\Phi$ forms the target (*N x 1)* magnetization vector, where $\Phi =(1,e^{i\varphi_{2}},e^{i\varphi_{3}},e^{i\varphi_{4}})$ is the excitation vector of the four transmit channels and *φ*_*i*_ is the relative phase to transmit channel 1. 3D single-channel E-fields together with density and conductivity matrices were extracted in order to compute the corresponding (4 x 4)-SAR-matrices [13, 25, 26]. Rapid SAR computations were conducted (SimOpTx, Vienna, Austria) for 10 g tissue mass averages [3]. With these (4 x 4)-SAR-matrices for both simulation models (Duke and Ella), a merit function *f*(**Φ**) was constructed to optimize B_1_^+^-homogeneity versus B_1_^+^-efficiency and local SAR_10g_:

$$f(\Phi )=\frac{std(\left|B_{1}^{+}(\Phi )\right|)}{mean(\left|B_{1}^{+}(\Phi )\right|)}-\beta *\frac{1}{\sqrt{\max\left({SAR}_{10g}^{Duke}(\Phi ),{SAR}_{10g}^{Ella}(\Phi )\right)}}*\frac{MOS(B_{1}^{+}(\Phi ))}{SOM(B_{1}^{+}(\Phi ))}$$ (1)

$$\begin{array}{ccc} \overbrace{B_{1}^{+}-homogeneity} & \;\overbrace{local\;SAR_{10g}} & \;\overbrace{B_{1}^{+}-efficiency} \end{array}$$

with MOS/SOM denoting the magnitude of sum/sum of magnitudes and a weighting factor *β*, which balances B_1_^+^-homogeneity (first term) with local SAR_10g_ and B_1_^+^-efficiency (second term). When setting the weighting factor *β* = 1, **Φ**_2_ minimizes this merit function.

As part of the validation of the phase optimization an isotropic raster search was performed using phase increments of 5° to examine each sub-term with varying β. For β ≥ 0.6 a balance between B_1_^+^-homogeneity, B_1_^+^-efficiency and SAR was observed, meaning that the phase setting to reach this balance stays unchanged. The TX phase setting deduced from the raster search (0°,-270°,-145°,-25°) and from the optimization procedure (0°,-271°,-142°,-25°) were equivalent (within a 3° variance), demonstrating that the optimization has indeed converged. **Φ**_2_: *φ*_1_ = 0°, *φ*_2_ = −271°, *φ*_3_ = −142°, *φ*_4_ = −25°

Phase setting **Φ**_**3**_. The procedure to obtain phase setting **Φ**_3_ was identical to **Φ**_2_, except that the target magnetization was restricted to an apical short-axis view (SAX) of the voxel model Duke. Thus with **Φ**_3_ the 4TX/4RX RF coil achieves a uniform and efficient excitation of an apical SAX. **Φ**_3_: *φ*_1_ = 0°, *φ*_2_ = −272°, *φ*_3_ = −60°, *φ*_4_ = −326°

### Validation of EMF Simulations in Phantom Experiments

To validate the EMF simulations of the body RF coil and the 4TX/RX RF coil, B_1_^+^-mapping was performed using a home-built torso phantom (dimensions H-F: 45 cm, L-R: 35 cm, A-P: 23 cm). The phantom was filled with a mixture of distilled water, copper sulfate (CuSO_4_, 0.73 g/l) and sodium chloride (NaCl, 3.3 g/l) to resemble electromagnetic tissue properties at 3T. Agarose (C_12_H_18_O_9_, 20g/l) was used as a gelling agent. The resulting electromagnetic properties (σ = 0.74 S/m, ε_r_ = 79.6) determined by complex impedance measurement were incorporated into the EMF simulations.

Absolute B_1_^+^-distributions of the individual RF coil configurations derived from the EMF simulations were compared to maps of absolute B_1_^+^-values derived from measurements. RF transmission field mapping was conducted using a Bloch-Siegert implementation [27, 28] (spatial resolution: 3x3x6 mm³, 4.5 ms Fermi pulse, off-center frequency: 4 kHz, TR = 80 ms, TA = 16 s) employing double gradient echo acquisitions (TE_1_/TE_2_ = 7.3/9.7 ms) to enable static magnetic field (B_0_) mapping. B_1_^+^-mapping was done offline using Bloch simulations in Matlab (MathWorks, Natick, MA, USA) considering B_0_ nonuniformities. To compare simulation and measurement on a pixel to pixel basis, the simulated B_1_^+^-fields of a mid-axial slice through the phantom were interpolated to the measured spatial grid and absolute difference maps were computed.

### RF Power and SAR Limits for the Volunteer Study

In order to compare the transmission regimes under investigation, the CMR examinations were driven on the RF coil-dependent SAR limit for each transmission regime. For a constant pulse duration and TR, SAR scales quadratically with the applied flip angle, which is directly proportional to the B_1_^+^-efficiency:

$$SAR\propto P_{in}\propto B_{1}^{+2}\propto {FA}^{2}$$ (2)

with the RF input power *P*_*in*_, the excitation field *B*_*1*_^*+*^ and the flip angle *FA*. For the transmission regimes using the large volume body RF coil the SAR limits are 2 W/kg for a 6 min and 4 W/kg for a 10 s averaging time [2]. For local cardiac transmit RF coil arrays local SAR_10g_ limits are 10 W/kg for a 6 min and 20 W/kg for a 10 s average. In clinical practice cardiac MR examinations are commonly conducted with breath hold acquisitions to eliminate respiratory motion artifacts. Clinically acceptable breath hold durations are kept in a range of approximately 10–15 s so that the 10 s averaging time SAR limit commonly applies for CMR.

Since the induced SAR per RF input power significantly differs between the BC/4RX and the 4TX/4RX regime and since local SAR_10g_ averaged over 10 s confines the applicable flip angle for breath-held cardiac MR protocols, the relevant metric to compare the transmission regimes was chosen to be

$$B_{1}^{+}\;@SAR\;limit=\frac{\left|B_{1}^{+}\right|}{\sqrt{P_{in}}}*\sqrt{\frac{SAR\;limit}{k}}={|B}_{1}^{+}|*\sqrt{\frac{SAR\;limit}{{max(SAR}_{simulation})}}$$ (3)

*k* = max(SAR_simulation_)/*P*_in_, *P*_*in*_ = *P*_*forward*_−*P*_*reflected*_, SAR_simulation_ and *B*_*1*_^*+*^ were retrieved from the EMF simulations. B_1_^+^@SAR limit is proportional to the maximum achievable flip angle *FA* within the regulatory limits [2]: $FA\propto \;B_{1}^{+}@SAR\;limit$.

It has been shown that local SAR_10g_ is a more restrictive measure than whole-body SAR [29, 30]. Consequently for the body RF coil transmission regime an additional protocol with a corrected flip angle *FA’* was acquired, which assumes local SAR_10g_ limits for the body RF coil according to:

$${FA}^{\prime }=FA*\delta$$ (4)

$$\delta =\sqrt{\frac{local\;SAR\;limit}{whole–body\;SAR\;limit}}*\sqrt{\;\frac{{whole–body\;SAR}_{Simulation}}{max(local\;{SAR}_{Simulation})}}\approx 0.7$$ (5)

The 1^st^ term is given by the IEC guidelines [2], whereas the 2^nd^ term was deduced from EMF simulations. Therefore, *FA’* reflects the maximal achievable flip angle of the body RF coil, if local instead of whole-body SAR limits were applied. As an additional safety margin, the entire study was performed in “normal operating mode” according to the IEC guidelines versus the less restricting “first/second level controlled operating modes”.

### Cardiac MR Volunteer Study

Prior to the volunteer study the four-channel transmit RF coil array underwent thorough safety assessment in line with IEC [2] which was evaluated and duly approved for implementation in clinical studies following certification by a notified body. For the in vivo feasibility study twelve healthy volunteers (age: 43±14 years, BMI: 25±3 kg/m², 8 males) without known history of cardiac diseases underwent CMR at 3T for all RF hardware configurations. This study was approved by the local ethics committee (registration number EA1/151/10, Charité—University Medicine, Berlin, Germany) and conducted according to the principles expressed in the Declaration of Helsinki [31]. Informed written consent was obtained from each volunteer prior to the study. All subjects were in normofrequent sinus rhythm (heart rate 75±12 bpm).

For in vivo transmission field mapping a 2D Bloch-Siegert technique [27, 32] was applied: spatial resolution: 5.3x5.3x6 mm³, 4.5 ms Fermi pulse, off-center frequency: 4 kHz, TE/TR = 7.3/80 ms, TA = 15 s. For each subject and transmission regime B_1_^+^-maps were acquired for a standard four-chamber (4CV), three-chamber (3CV), two-chamber (2CV) and a short-axis view (SAX) of the heart in end-diastole to eliminate blood flow and motion artifacts. Based on the B_1_^+^-maps (actual) flip angle maps were calculated.

Functional imaging of the heart was performed according to a standardized clinical CMR protocol [33–35] using 2D steady-state free precession (SSFP) CINE: spatial resolution: 1.8x1.8x6 mm³, FoV: 340x340 mm^2^, *τ*_*RF*_ = 1.2 *ms*, RF pulse duration-bandwidth_TX_-product = 1.6, duty cycle = 38%, 30 cardiac phases, TA = 15–18 s (within one breath hold), TE/TR = 1.4/3.2 ms, bandwidth_RX_ = 1002 Hz/pixel. No filtering such as zero-filling was performed. For each subject and transmission regime 2D SSFP CINE images were acquired in 4CV, 3CV, 2CV and SAX orientation of the heart.

Due to the short TR, high flip angle and high duty cycle 2D SSFP imaging techniques are SAR confined at 3T which renders it an ideal candidate for the assessment of the SAR performance of the transmission regimes under investigation. To alter the RF duty cycle and the RF power deposition of the 2D SSFP CINE technique, without modifying the RF pulse duration or shape, the repetition time TR was changed by varying the receiver bandwidth BW_RX_ from 1532 Hz/pixel to 130 Hz/pixel enabling a TR between 3.1 ms and 8.3 ms, while keeping all other imaging parameters constant with the exception of SNR which scales with 1/√BW_RX_.

For the clinical assessment of each transmission regime left ventricular (LV) chamber quantification was performed with 2D SSFP CINE. For this purpose a parallel stack of short-axis views covering the heart from apex to base with an inter-slice gap of 3 mm was acquired using the imaging parameters outlined in the previous paragraph. For each subject and transmission regime LV end-diastolic volume (LV EDV), LV end-systolic volume (LV ESV), LV ejection fraction (LV EF) and LV myocardial mass (LV mass) were obtained using CMR42 (Circle Cardiovascular Imaging, Calgary, Canada).

For all in vivo CMR examinations cardiac gating and triggering was achieved with an MR stethoscope [36–39] (EasyACT, MRI.TOOLS GmbH, Berlin, Germany).

## Results

### SAR Simulations

**Fig 2:**
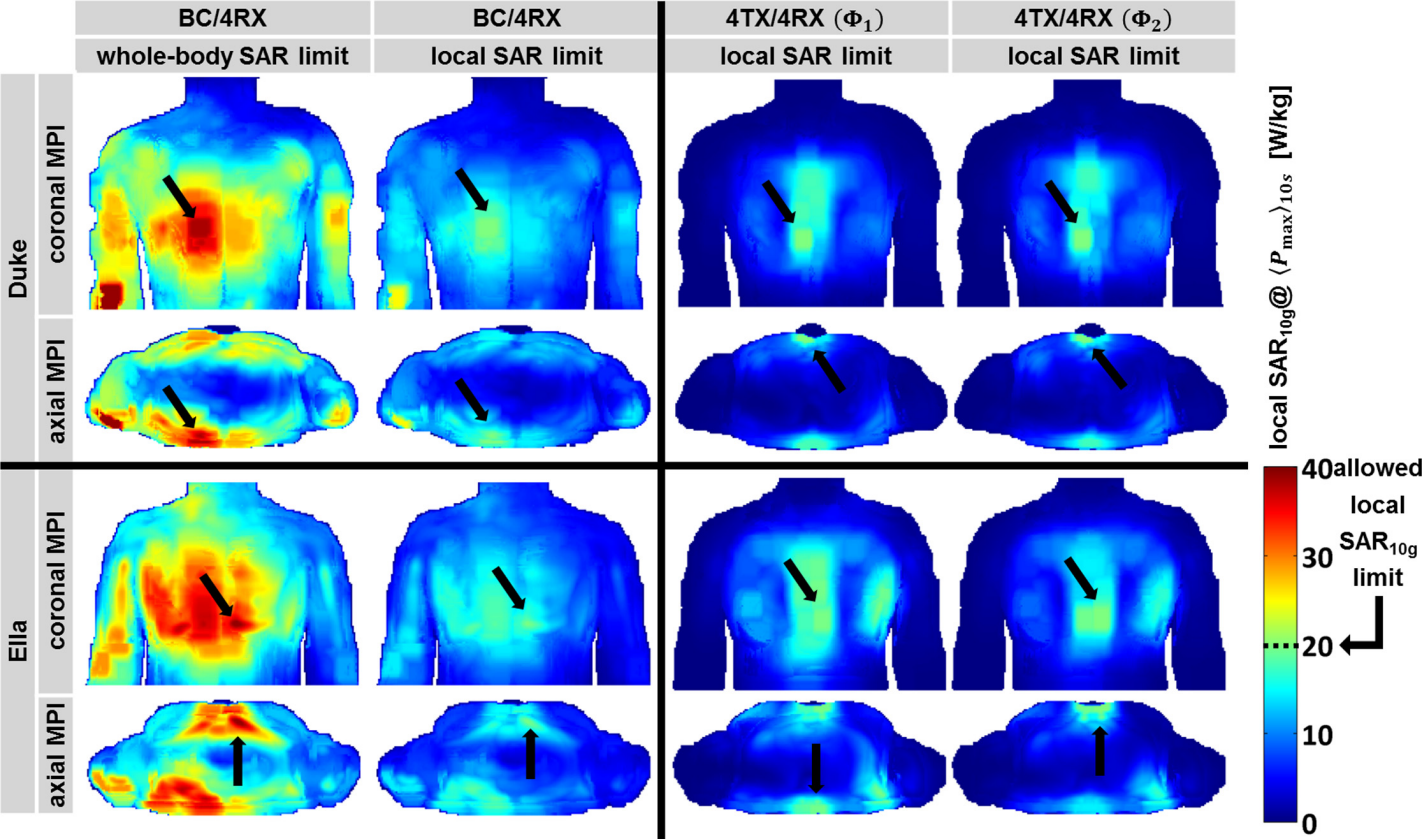
Simulated local SAR_10g_ distributions at maximum applicable input power. Maximum projection images for voxel model Duke (top) and Ella (bottom) in coronal (1^st^ and 3^rd^ row) and axial orientation (2^nd^ and 4^th^ row) are shown. Formations of SAR hotspots can be seen for every transmission setup, with the dominating SAR hotspots being marked by arrows. Note: At the elbow twice the SAR is allowed to avoid a bias in favor of the 4TX/4RX configuration. Immoderate whole-body SAR limits lead to exceedance of local SAR limits (1^st^ column). For 4TX/4RX both transmit phase settings Φ_1_ (3^rd^ column) and Φ_2_ (4^th^ column) yield a local SAR_10g_ hotspot located underneath the shared middle conductor of the loop elements, where the currents can add up and the distance between the RF coil array and the body is minimal (1 cm/2 cm for the anterior/posterior part).

EMF simulations were performed to derive SAR distributions across the upper torso for all transmission regimes. Fig 2 surveys maximum projection images of local SAR_10g_ for maximum allowed RF input power (see Table 1) for the human voxel models Duke and Ella. For BC/4RX RF power deposition was found across the entire upper torso with local SAR_10g_ hotspots being located around the right elbow (Duke) and the anterior chest (Ella) (Fig 2). Whole-body SAR was found to be 0.0068 W/kg for Duke and Ella for an input power of 1 W. When applying the IEC limits for whole-body SAR the total averaged (10 s) input power was found to be 590 W. When using the local SAR_10g_ limits, the RF power needed to be reduced by a factor of ~2 to stay within the IEC limits as summarized in Table 1.

**Table 1:** SAR and B_1_^+^-values derived from EMF simulation.

used transmission setup		BC/4RX						4TX/4RX (Φ_1_)		4TX/4RX (Φ_2_)		4TX/4RX (Φ_3_)	
applied SAR limit		whole-body SAR		partial-body SAR		local SAR_10g_		local SAR_10g_		local SAR_10g_		local SAR_10g_	
(within 10 s)	[unit]	4 W/kg		4–20 W/kg		20 W/kg		20 W/kg		20 W/kg		20 W/kg	
*k* = SAR/*P*_in_	[1/kg]	0.0068	0.0068	0.014	0.017	0.065	0.069	0.43	0.38	0.38	0.40	0.39	x
						(0.082^#^)							
⟨*P*_max_⟩_10*s*_	[W]	590	590	890	840	310	290	47	52	53	50	51	x
$std(\left|B_{1}^{+}\right|)/mean(\left|B_{1}^{+}\right|)$	[%]			15	16			32	25	30	19	38 (21^§^)	x
$MOS/SOM\;(B_{1}^{+})$	[%]							81	83	80	82	80 (91^§^)	x
$mean(\left|B_{1}^{+}\right|/\sqrt{P_{in}})$	$\left[\mu T/\sqrt{kW}\right]$			5.2	6.5			13	16	12	16	12 (15^§^)	x
$mean(\left|B_{1}^{+}\right|)\;@\;SAR\;limit$	[μT]	4.0	5.0	4.9	5.9	2.9	3.5	2.7	3.7	2.9	3.6	2.8 (3.3^§^)	x

The left/right value of each cell is derived from simulation with voxel model Duke/Ella. For SAR assessment all voxels of the simulation models were used. Please note, that the SAR hotspot at Duke’s elbow (^#^) is not limiting the applicable input power as twice the local SAR is allowed at the extremities. The 10 s averaged maximum RF input power to reach the SAR limit given by the IEC guideline is denoted by ⟨P_max_⟩_10s_. For B_1_^+^-evaluation only voxels covering the voxel model’s heart (3D) were considered. The metric std/mean (coefficient of variation) and MOS/SOM of the transmit field reflects the B_1_^+^-homogeneity and -efficiency in the target region. The resulting B_1_^+^-field inside the heart at the maximum input power is given by mean(|*B*_1_^+^|)@SAR limit, which is proportional to the maximum achievable flip angle. The ratio between |*B*_1_^+^|@local to whole-body SAR is approx. *δ* ≈ 0.7. For **Φ**_3_ voxel model Duke was simulated only. When calculating the B_1_^+^ inside an apical SAX (2D), which was subject for generating **Φ**_3_, the values in parentheses apply.

^#)^ at Duke’s elbow

^§)^ only voxels within an apical SAX (2D)

Our findings showed that the whole-body SAR limit of 4 W/kg (within 10 s) puts less RF power deposition constraints onto the body RF coil transmission regime than the local SAR_10g_ limit of 20 W/kg (within 10 s). To highlight this difference, 2^nd^ column of Fig 2 outlines the SAR_10g_@⟨P_max_⟩_10s_ when applying the local SAR_10g_ limits for body RF coil transmission. Different regulatory limits for trunk (20 W/kg within 10 s) and extremities (40 W/kg within 10 s) were considered (e.g. Duke’s elbow).

For 4TX/4RX using phase settings **Φ**_1_ and **Φ**_2_ local SAR_10g_ was observed to be largest in upper torso areas where the RF coil array conforms best to the upper chest and where the distance between the RF coil and the chest wall is lowest. Other body regions, such as the head, abdomen or arms experienced almost no RF power deposition. Phase setting **Φ**_1_, which was tailored for B_1_^+^-efficiency across the heart without considering local SAR_10g_, shows a maximum local SAR_10g_–hotspot of 0.43 W/kg for Duke and 0.38 W/kg for Ella (P_in_ = 1 W) for a region close to the sternum. Phase setting **Φ**_2_ which includes local SAR_10g_ optimization over B_1_^+^-efficiency, local SAR_10g_ assessment yielded a maximum local SAR_10g_ of 0.38 W/kg for Duke and 0.40 W/kg for Ella (P_in_ = 1 W). For the in vivo measurements the maximum applied input power ⟨P_max_⟩_10s_ was 47 W, 50 W and 51 W for 4TX/RX driven with the phase settings **Φ**_1_, **Φ**_2_ and **Φ**_3_.

### B_1_^+^ Simulations

**Fig 3:**
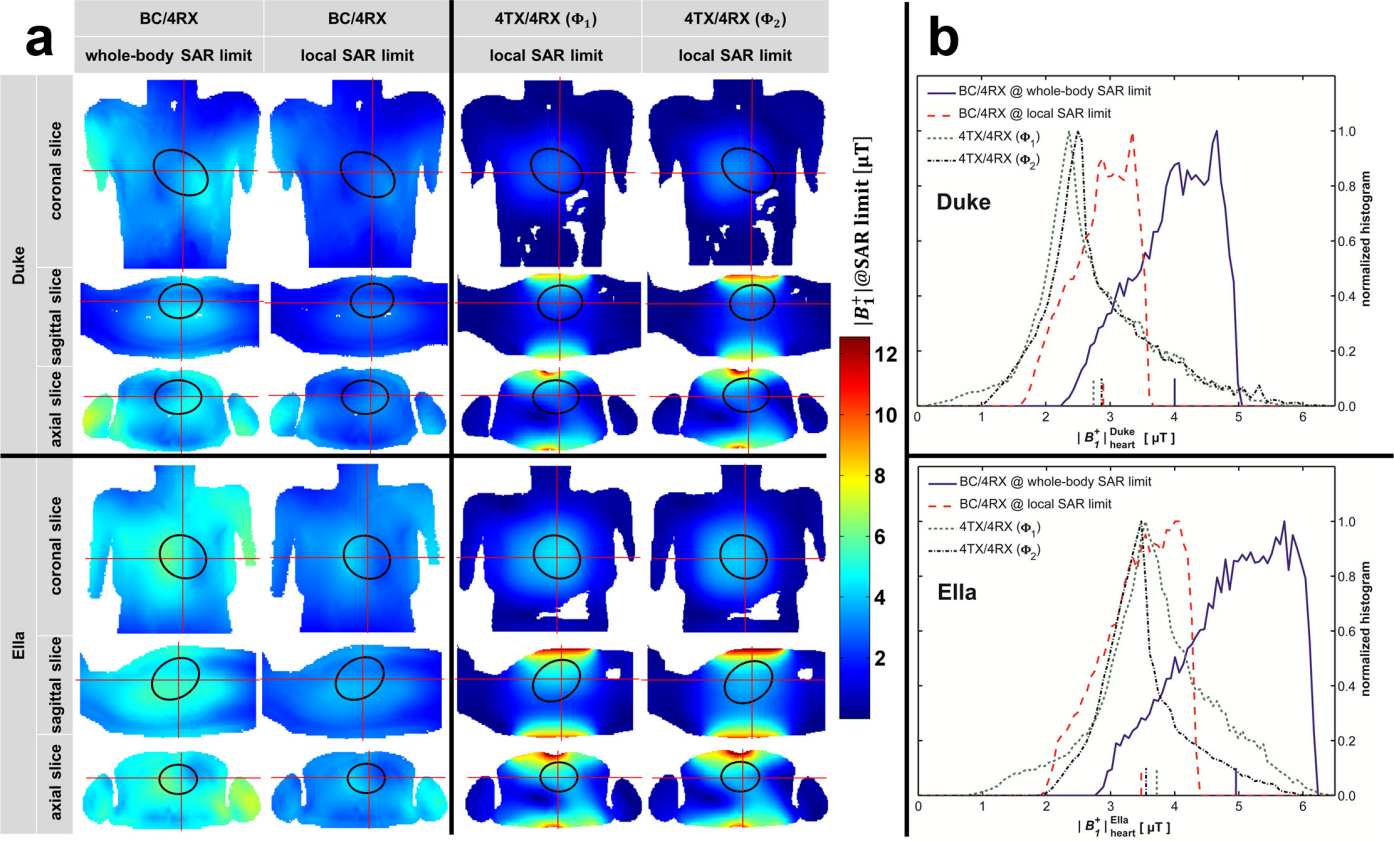
Simulated excitation fields at specified SAR limits. (a) B_1_^+^-fields for Duke (top) and Ella (bottom) are derived from EMF simulations for all transmission setups. To guide the eye the orthogonal slices and the borders of the heart are highlighted. 1^st^ column: BC/4RX scaled to whole-body SAR limit. 2^nd^ column: BC/4RX scaled to local SAR_10g_ limit. 3^rd^ and 4^th^ column: Transmission with 4TX/4RX RF coil (Φ_1_ and Φ_2_) scaled to local SAR_10g_ limit. The data shown in column 1, 3 and 4 reflect the maximum achievable excitation fields to stay within the safety limits governed by the IEC guidelines [2]. (b) Normalized histograms of the simulated excitation fields obtained for all voxels covering the heart of the human voxel model Duke (top) and Ella (bottom). The mean values of the B_1_^+^-distributions are added as colored tick marks on the x-axes. The width of the curves reflects the B_1_^+^-homogeneity within the heart. The red and blue curves are shifted horizontally by the factor *δ* ≈ 0.7, which represents the RF input power difference of the body RF coil when operating at whole-body and local SAR limit.

Table 1 shows that the induced SAR/P_in_ and B_1_^+^/√P_in_ significantly differ between the body and the local RF coil regime. B_1_^+^@SAR limit (see Eq 3) sets both quantities in relation and thus makes the different RF coil regimes comparable. Fig 3A illustrates the B_1_^+^-distributions at the specified SAR limits for a coronal, sagittal and axial slice through Duke’s and Ella’s heart.

The B_1_^+^-pattern obtained for whole-body and local SAR limits (1^st^ and 2^nd^ column in Fig 3A) are identical but the mean value of B_1_^+^@SAR limit differs by the factor *δ*≈0.7 (Eq 5) in favor of the whole-body SAR limit. The simulated transmission fields obtained for 4TX/4RX are depicted in Fig 3A for the phase settings **Φ**_1_ and **Φ**_2_. The B_1_^+^-homogeneity across the heart found for **Φ**_2_ is superior to that observed for **Φ**_1_. In particular, the coronal view obtained for Duke and the sagittal view derived from Ella revealed less regions of low B_1_^+^ across the heart for **Φ**_2_.

These findings are highlighted by Fig 3B, which shows B_1_^+^ histograms for all voxels covering Duke’s and Ella’s heart. The right tails of the histograms obtained for 4TX/4RX with **Φ**_1_ and **Φ**_2_ relate to regions close to the apex. The left tails of the histograms are related the basal region of the heart and are significantly reduced for **Φ**_2_ compared to **Φ**_1_. The B_1_^+^-coefficient of variation (standard deviation/mean) obtained for the **Φ**_2_ histogram is lower than that of the **Φ**_1_ histogram, which indicates an improved B_1_^+^-homogeneity of **Φ**_2_ versus **Φ**_1_. The mean values of B_1_^+^@SAR limit, a measure of B_1_^+^-efficiency (ticks on x-axes in Fig 3B) are summarized in Table 1. The difference between the B_1_^+^ histogram obtained for the body RF coil at whole-body SAR limit and at local SAR_10g_ limit (red and blue curves) is due to the factor *δ*≈0.7, which translates into a horizontal shift of the B_1_^+^ histograms.

The loading of the transmit RF coil, i.e. the used voxel model Duke or Ella, was found to have an influence on the RF coil performance. When changing the loads from Duke to Ella the B_1_^+^@SAR limit increases by 21% for the body RF coil, 37% for 4TX/4RX RF coil (**Φ**_1_) and 24% for 4TX/4RX RF coil (**Φ**_2_).

### Validation of EMF Simulations in Phantom Experiments

**Fig 4:**
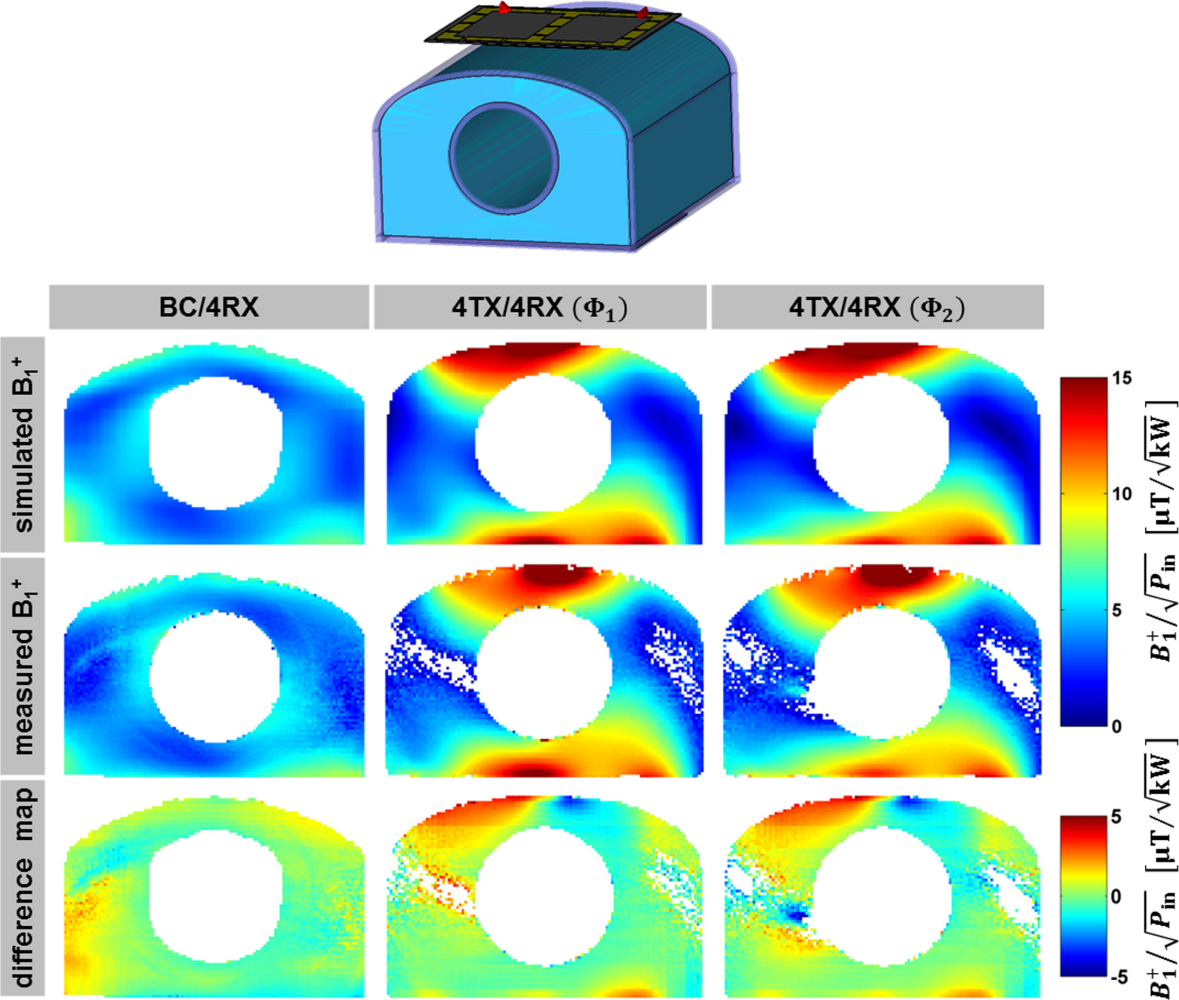
Validation of EMF simulations. Comparison of simulated (1^st^ row) and measured (2^nd^ row) B_1_^+^-maps of a mid-axial slice through a torso phantom (top) filled with a uniform myocardial tissue mimicking solution. Regions with angle-to-noise-ratios lower than 1% were discarded using thresholding. 3^rd^ row: Absolute difference maps (B_1_^+^_simulation_ - B_1_^+^_measurement_) demonstrating a good agreement between simulations and experiments.

The EMF simulations were validated in phantom experiments. Fig 4 illustrates the simulated and measured B_1_^+^-field distributions together with absolute difference maps for a mid-axial slice of the upper torso phantom for BC/4RX and 4TX/4RX (**Φ**_1_ and **Φ**_2_). Regions with low angle-to-noise-ratios (<1%) were excluded from the comparison using a threshold. For the body RF coil the simulated and measured B_1_^+^-field (mean±std) was 4.4±1.2 $\mu T/\sqrt{kW}$ and 4.3±1.2 $\mu T/\sqrt{kW}$. For the local 4TX/4RX RF coil (**Φ**_1_) the simulated and measured excitation fields yielded 6.7±3.8 $\mu T/\sqrt{kW}$ and 6.5±3.6 $\mu T/\sqrt{kW}$, while for **Φ**_2_ it was 6.6±3.9 $\mu T/\sqrt{kW}$ and 6.4±3.6 $\mu T/\sqrt{kW}$.

The interference pattern was found to be in good agreement, which demonstrates the validity of both RF coil models and supports the credibility of the SAR simulations.

### Cardiac MR Volunteer Study

**Fig 5:**
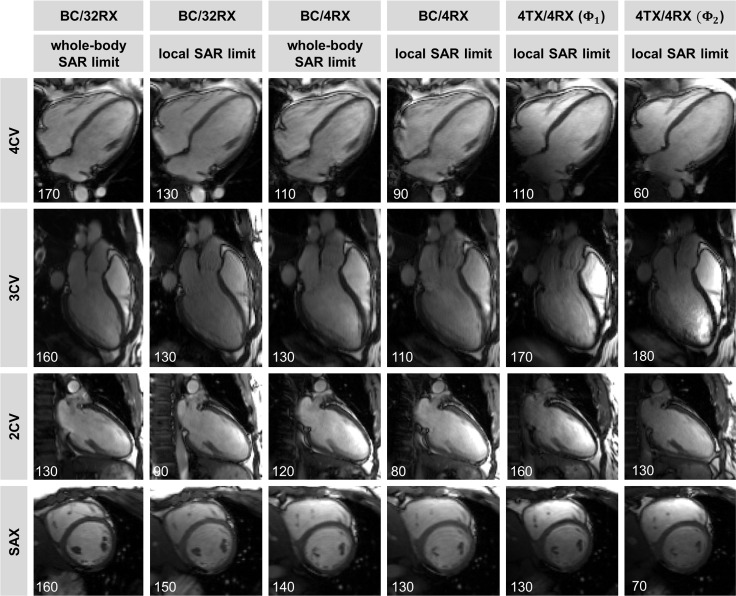
Cardiac images derived from 2D SSFP CINE. Shown are end-diastolic phases of the cardiac cycle using standard cardiac views (denoted in the left line) of a healthy subject. The employed transmission regime is outlined on top of the figure for each column. Each image was windowed individually and its SNR within the heart is provided. Column 1, 3, 5 and 6 were acquired with the maximum flip angle allowed by SAR limits governed by the IEC guidelines [2]. Column 2 and 4 were derived by applying local SAR_10g_ limits for the body RF coil, which results in 30% reduced flip angle compared to whole-body SAR limit (1^st^ and 3^rd^ column). Please note the agreement in image quality obtained with the BC/4RX, BC/32RX and the 4TX/4RX RF coil configurations.

Fig 5 provides a synopsis of the CMR volunteer study using all transmission regimes. The repetition time TR was fixed and FA was increased until the SAR limit was reached for each transmission regime. No B_1_^+^ induced signal voids were found across the entire FoV even for 4TX/4RX transmission. For the 4TX/4RX RF coil a signal intensity gradient can be detected, which manifests itself in an enhanced signal in the right ventricle in the three-chamber view due to its proximity to the surface coil array. The difference between cardiac images obtained for the body RF coil at whole-body SAR limit and at local SAR_10g_ limit is due to the flip angle reduction of δ≈0.7. This change in the transmission field was found to have minor effect on the blood-myocardium contrast due to the relatively flat signal versus flip angle curve of the SSFP technique [35, 40].

**Fig 6:**
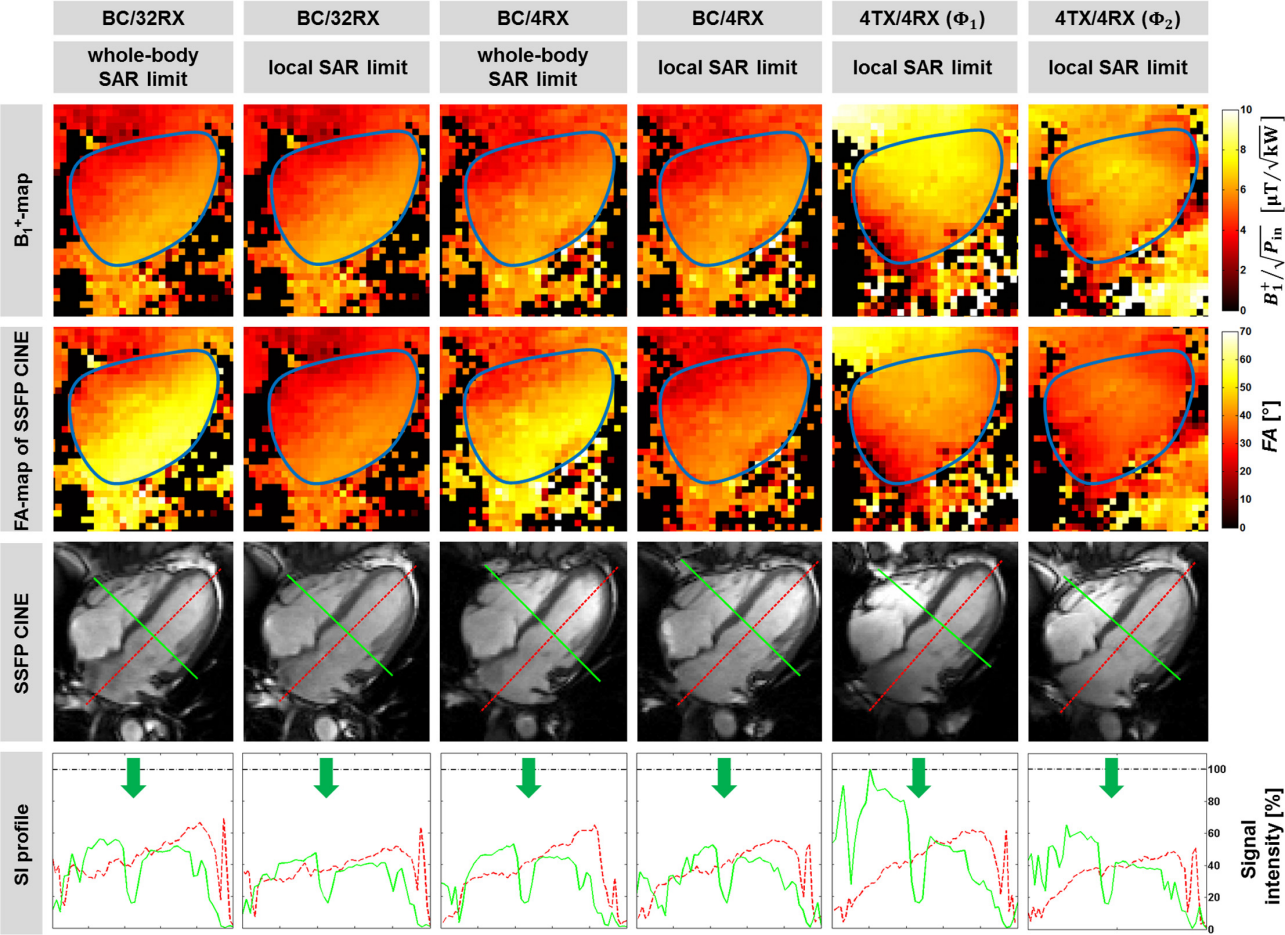
Quantitative analysis of 2D SSFP CINE images for a four-chamber view. Assessment of in vivo performance of the transmission regimes illustrated for a four-chamber view of the heart of a healthy subject. The employed transmission regimes are outlined on top of the figure for each column. 1^st^ row: Measured B_1_^+^-maps obtained with a 2D Bloch-Siegert technique. The borders of the heart are highlighted with a bold line. 2^nd^ row: Flip angle maps for SSFP CINE at specified SAR limit, which were derived from the B_1_^+^-maps. 3^rd^ row: 2D SSFP CINE images of the same four-chamber view obtained at end-diastole using maximum flip angles at the specified SAR limit (denoted on top). 4^th^ row: Normalized signal intensity profiles along the lines drawn through the four-chamber view shown above. The green arrows indicate the position of the septum. The 1^st^ and 3^rd^ column represents the maximal applicable flip angle allowed by the IEC guidelines, i.e. the whole-body SAR limit. The 2^nd^ and 4^th^ column show the situation if local SAR_10g_ limits were applied for the body RF coil. The data shown in the 5^th^ and 6^th^ column were acquired with the 4TX/4RX RF coil with phase setting **Φ**_1_ and **Φ**_2_ at local SAR_10g_ limits.

For quantification of the transmission fields in vivo B_1_^+^-maps were acquired and flip angle maps were calculated. Fig 6 shows a standard four-chamber view of a healthy female subject (34 years, BMI: 25 kg/m²) with the contours of the heart being highlighted. The transmission field and the flip angle across the heart were found to be most uniform for the 4TX/4RX regime driven with phase setting **Φ**_2_. This regime resulted in signal intensity profiles across the right and left ventricle which are competitive with those obtained for body RF coil transmission (Fig 6). Blood-myocardium contrast was approximately 70 (BC/32RX), 50 (BC/4RX) and 80 (4TX/4RX). This supports ample delineation of endo- and epicardial borders when using the 4TX/RX configuration.

**Fig 7:**
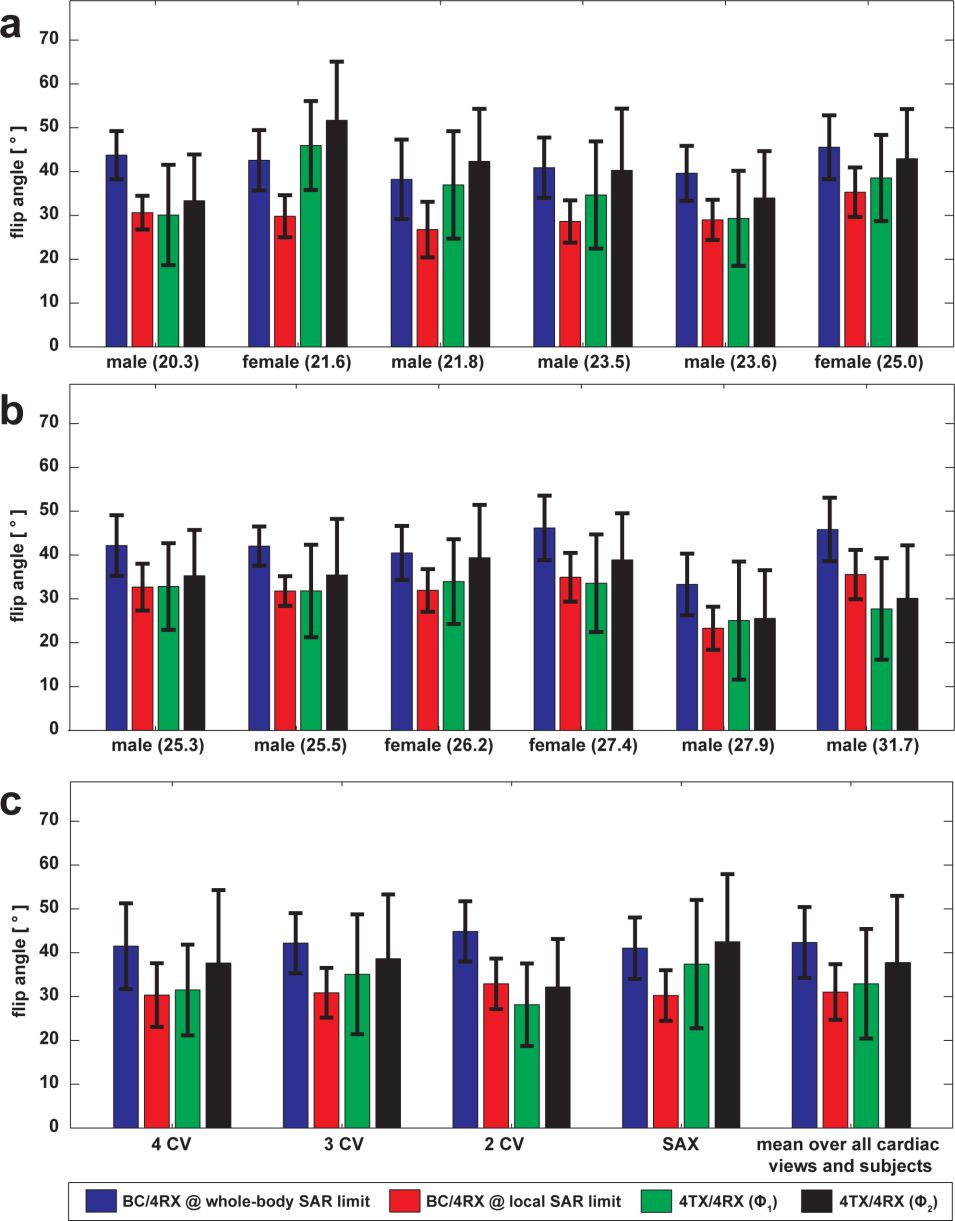
Analysis of achieved SSFP-flip angles inside the heart of the scanned cohort. The bar height reflects the mean FA thus the transmit efficiency. The error bars show the standard deviation thus the transmit homogeneity within the ROI. (a) and (b) The flip angle bars are grouped for each subject of a cohort of 12 subjects. Gender and BMI are provided. Averaging over the cardiac views (4CV, 3CV, 2CV, SAX) was performed. The data show the FA variability between individual subjects, which is minor for the body RF coil but more pronounced for the 4TX/4RX RF coil. (c) Mean flip angle resolved for different cardiac views. For each cardiac view FA data was averaged over all 12 subjects. The 4TX/4RX RF coil configuration yielded a B_1_^+^-efficiency which outperformed the body RF coil when operated at local SAR_10g_ limit for all standard cardiac views except the two-chamber view. The last bar group shows the flip angle averaged over all subjects and all cardiac views. The 4TX/4RX RF coil configuration using phase setting **Φ**_2_ yielded a B_1_^+^-efficiency which is superior to that obtained for phase setting **Φ**_1_ while having similar B_1_^+^-homogeneity.

A closer examination of the flip angle maps obtained for all subjects is summarized in Fig 7. Averaged over all subjects and cardiac views the mean±std of the flip angle were: 42°±8° for BC/4RX at whole-body SAR limit, 31°±6° for BC/4RX at local SAR_10g_ limit, 33°±12° for 4TX/4RX (**Φ**_1_) and 38°±15° for 4TX/4RX (**Φ**_2_). The inter-subject variation was slightly increased for the 4TX/4RX RF coil versus body RF coil transmission (compare Fig 7A and 7B). For cardiac mid-ventricular short-axes, four-chamber and three-chamber views the local 4TX/4RX RF configuration (**Φ**_2_) provided a mean flip angle which was superior to the body RF coil transmission with B_1_^+^@local SAR_10g_ limit (Fig 7C). For two-chamber views the body RF coil produced a higher mean flip angle versus the 4TX/4RX RF coil.

**Fig 8:**
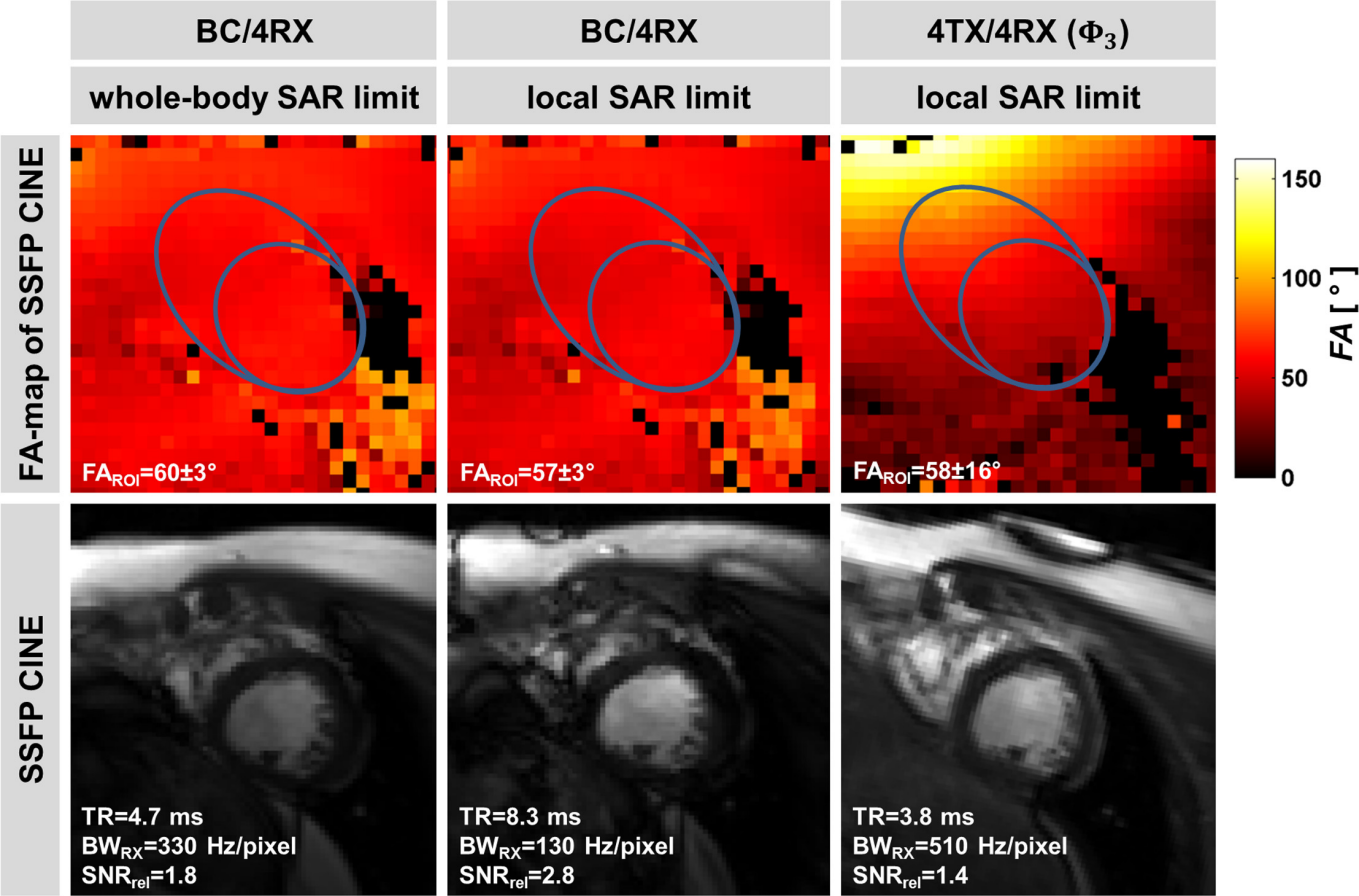
Effects of transmit efficiency on minimal TR_SSFP_. Shown are flip angle maps and 2D SSFP CINE images of an apical short axis view of the heart. Based on the FA-maps (top row) a target FA of 60° inside the ROI (blue contour) was set for the 2D SSFP CINE technique (bottom row). TR_SSFP_ was set to minimum so that SAR reached the denoted limits. When operating the body RF coil at whole-body/local SAR limit, the B_0_ pass band of 2D SSFP CINE exhibited a width of 212 Hz/120 Hz. Severe banding artifacts can be seen for BC/4RX @ local SAR limit. When using the 4TX/4RX RF coil (**Φ**_3_) a pass band of 263 Hz was achieved for 2D SSFP. This improvement helps to reduce SSFP related banding artifacts across the heart. The denoted SNR_rel_ is relative to the SNR obtained with the SSFP protocol (BW_RX_ = 1002 Hz/pixel) used for LV chamber quantification.

Next, based on the FA-maps shown in Fig 8 the mean flip angle FA_SSFP_ inside a ROI covering the heart in an apical SAX orientation was set to 60° and TR_SSFP_ was adjusted to reach the specified SAR limit. For these settings SNR_blood_ was superior to SNR_blood_ obtained for the cardiac views used for the LV chamber quantification study as outlined in Fig 8. Due to the efficiency of the 4TX/4RX setup (**Φ**_3_) (TR_min_ = 3.8 ms) a TR_min_ reduction of 19% or 54% was achieved versus body RF coil transmission at whole-body SAR limit (TR_min_ = 4.7 ms) or at local SAR_10g_ limit (TR_min_ = 8.3 ms). The TR_min_ shortening of the local 4TX/4RX RF coil helped to reduce SSFP banding artifacts and relaxed the constraints on the fidelity of volume-selective B_0_-shimming.

**Fig 9:**
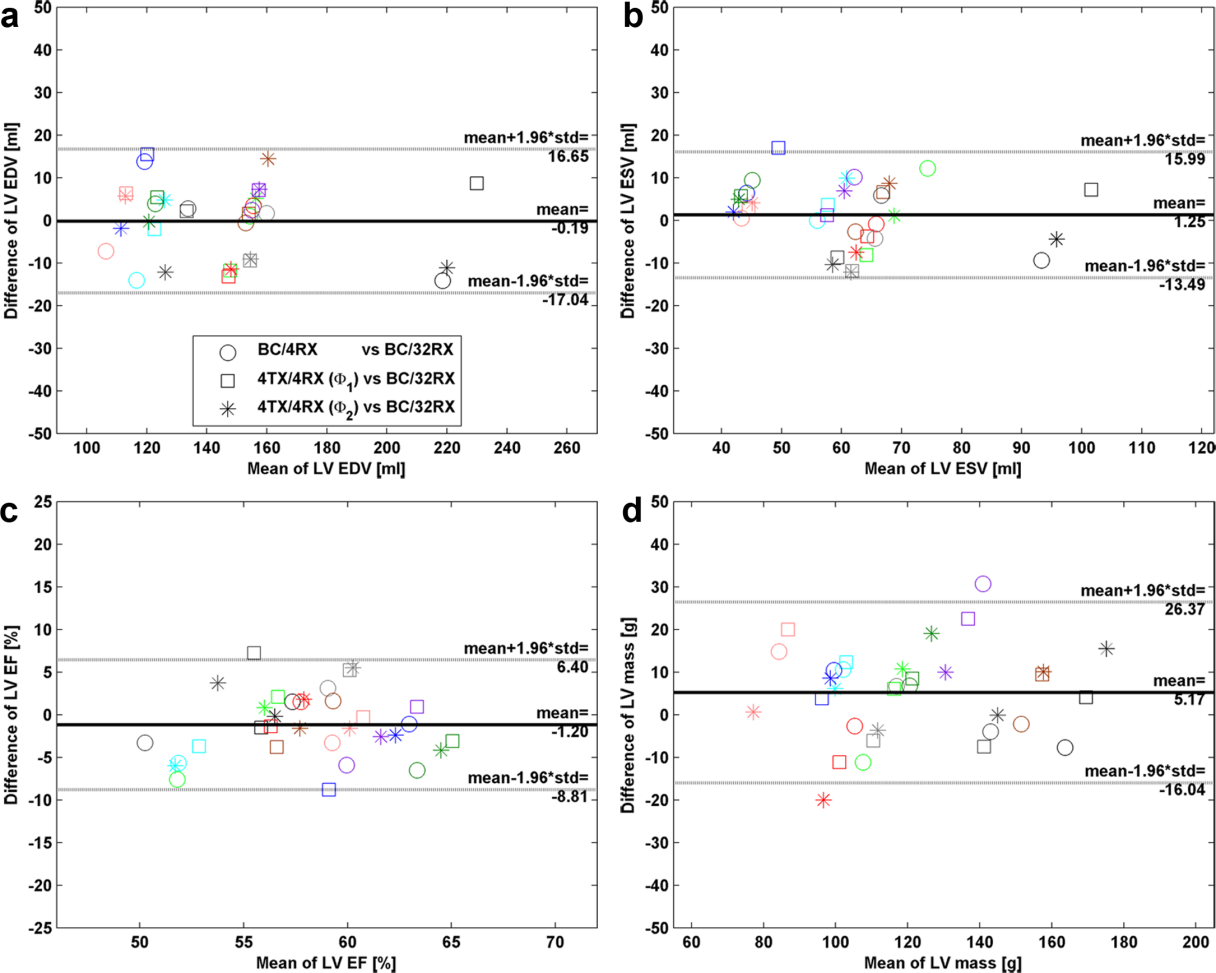
Effects of transmission regime on left ventricle quantification. Left ventricle (LV) cardiac chamber quantification in a cohort of 11 subjects obtained for all transmission regimes were examined by evaluating a stack of short axis views ranging from apex to base. Bland-Altman plots of (a) LV end-diastolic volume (EDV), (b) LV end-systolic volume (ESV), (c) LV ejection fraction (EF) and (d) LV mass. No statistically significant differences were found between the reference (BC/32RX) and the BC/4RX setup (circles), the 4TX/4RX with phase setting **Φ**_1_ (squares) or the 4TX/4RX with phase setting **Φ**_2_ (stars). Data points of the same subject were marked with identical colors.

The outcome of the cardiac chamber quantification is summarized in Fig 9. In the Bland-Altman analysis the clinical standard setup (BC/32RX) was used as a reference. The values for LV EDV, ESV, EF and mass obtained with the other transmission regimes (BC/4RX and 4TX/4RX) showed no statistically significant differences.

## Discussion

Our simulations and experiments demonstrate that CMR and cardiac chamber quantification at 3T using local four-channel surface RF coil transmission is competitive when benchmarked against the today’s clinical CMR practice of large volume body RF coil transmission. The implications feed into a broad range of applications which stand to benefit from local surface RF coil transmission. CMR of patients equipped with passively conducting implants that constitute an SAR governed contraindication for cardiac MR in case of body RF coil transmission presents an application where local surface RF coil transmission provides a vital alternative over RF body coil excitation. Another example includes interventional CMR employing imaging guided navigation of conducting catheters. Our results showed that the B_1_^+^-efficiency benefits of a local transmission coil array versus body coil transmission can be translated into a reduction in the repetition time. This speed gain is conceptually appealing for the pursuit of rapid imaging that supports real time assessment of the heart rather than relying on the interpolation of data acquired during multiple synchronized heartbeats commonly used in today’s CINE acquisitions.

Although whole-body SAR is used in clinical practice to limit the RF power of a body RF coil, local SAR_10g_ limits are more restrictive. When applying the maximum power to reach the whole-body SAR limit with the body RF coil (4 W/kg within 10 s) the local SAR_10g_ limit (20 W/kg within 10 s) is already exceeded by 90%. This indicates that the local SAR_10g_ limits are rather conservative. These results accord with previous reports for RF transmission of the head [29] and the body [30] and underline the relevance of using local SAR_10g_ limits for a fair comparison between body RF coil and local surface RF coil transmission.

In our study we performed static B_1_^+^-shimming including local SAR_10g_ considerations for the local surface RF coil. By employing a SAR-matrix formalism [13] and a compression algorithm [26] the SAR calculation is fast enough to enable reasonable computation times. This allows for an optimization function that balances local SAR_10g_, B_1_^+^-homogeneity and -efficiency. With this B_1_^+^-shimming approach (**Φ**_2_) larger flip angles with higher B_1_^+^-homogeneity can be achieved versus B_1_^+^-shimming (**Φ**_1_) without local SAR_10g_ considerations. The short distance to the human chest renders the local transmit surface RF coil more efficient than the body RF coil in terms of transmit efficiency $B_{1}^{+}/\sqrt{P_{in}}$ as well as $B_{1}^{+}/\sqrt{local\;SAR}$. This behavior resulted in an input power of P_in_≈170 W for a flip angle of 90° (1 ms rectangular pulse). To accomplish the same flip angle for body RF coil transmission this input power needed to be increased by a factor ≈6 (P_in_≈1000 W). As a consequence, current clinical CMR at 3T is driven by 35 kW RFPAs for body RF coil transmission, whereas an 8 kW RFPA is sufficient for local TX/RX RF coil arrays.

Our in vivo B_1_^+^-mapping results showed that the mean flip angle across the heart is higher for the local surface RF coil array versus body RF coil transmission when the same SAR limits are employed. High transmission efficiency is clinically relevant for CMR, where the constraints dictated by cardiac and respiratory motion limit the viable window of data acquisition so that the use of rapid imaging techniques such as SSFP are mandatory for cardiac chamber quantification.

Our findings have practical implications including MR safety. Body RF coil transmission induces RF power deposition in a large volume including body regions far away from the ROI. This is of clinical relevance for cardiac patients with hip replacement prosthesis (clinical prevalence in an older population of up to 5% [41]) and other abdominal or peripheral implants (for example vena cava filter, clinical prevalence in an older population of up to 4% [42]) which constitute an RF-heating related contraindication if body RF coil transmission is used [43]. Local transmit RF coils dedicated for CMR restrict power deposition primarily to the upper chest and hence permit inclusion of these patients into CMR examinations. Interventional CMR examinations are another application which might benefit from multi-channel RF surface coil rather than body RF coil transmission. The degrees of freedom of a multi-channel surface coil array can be used to generate electric-field-reduced zones without significantly altering the transmit sensitivity [44]. This approach can be tailored to create “null mode” excitations that induce minimal RF current in elongated conductors such as pacemaker leads and conductive guidewires used for interventional procedures, thereby decreasing the RF heating hazard, while still allowing imaging of the surrounding volume [45]. Because implant heating is directly related to E-field distribution, implant-friendly multi-channel RF coil arrays can be designed following this approach. A recently proposed generalized but analytical approach went even further and provided a novel design criterion for multi-channel transmit RF coil arrays based upon RF heating assessment of passive electrically conductive implants including coronary stents and interventional catheters [46].

The limited number of elements used for the local TX/RX RF coil array is an acknowledged limitation of this feasibility study along with the application of RF phase control only. Also, a gradient in signal intensity ranging from the body surface to the center of the upper torso persists for the local TX/RX RF coil configuration. This non-uniformity is due to destructive B_1_^+^-interference. It also embodies the transmit and receive sensitivity profile of the array of surface coils. Supporting exquisite control over the electromagnetic fields by modulating amplitude and phase used for excitation of each transmit channel independently together with boosting the number of transmission channels would increase the degrees of freedom. An increased number of RF transmit channels enhances B_1_^+^-homogeneity and image quality, as previously demonstrated for 7T TX/RX cardiac optimized RF coil arrays [5–8]. Since the current clinical standard at 3T commonly employs a 32-channel receive surface coil array, the clinical approach would not be hampered but rather improved by including transmit/receive functionality for cardiac optimized RF surface coil arrays. If advanced carefully through joint technical developments and clinical studies this approach bears the potential for paving the way toward making the body RF coil obsolete for cardiac MR. This would significantly benefit patient comfort by increasing the effective bore size from 70 cm diameter to 91 cm. It would also save major manufacturing costs so far allocated for the body RF coil.

The use of multi-channel TX/RX RF surface coil arrays would support subject specific RF shimming to improve B_1_^+^-homogeneity as compared to single-channel body RF coil transmission [47, 48]. Possible RF shimming benefits were also reported for multi-channel body RF coil transmission [49, 50]. This has been recently confirmed for parallel transmission with a range of body RF coil arrays, which all outperformed the conventional birdcage body RF coil in all metrics except peak and average power efficiency [51]. The question of the best distance between RF coil and subject in terms of RF power requirements and local SAR deposition was subject to [52]. They report an optimal distance between object and RF coil consisting of 12 to 20 transceiver elements of approx. 6 cm, which is in favor of local surface coil arrays rather than whole-body RF coils. Unlike the majority of the installed base of 3T scanners which are commonly equipped with body coils with only one feeding port and thus only one excitation pattern, a recently introduced two-channel RF body coil affords zoomed excitations [48]. This approach might help to limit RF power deposition away from the area of interest. Yet zoomed imaging with parallel transmission [53] requires dedicated RF pulses which are longer than conventional RF pulses used for 2D slice excitation and hence provide a time penalty. This drawback might be challenging if not prohibiting for short TR acquisitions needed for cardiac MR to balance the competing constraints of spatial and temporal resolution.

To conclude, pursuing local surface RF coil arrays for transmission in cardiac MR is a conceptually appealing alternative to body RF coil transmission. While improved local transmit RF coil designs for 3T still need to be investigated, the clinical potential and implications derived from physical and technical considerations are convincing to put further weight behind these developments.
